# Canine-Based Strategies for Prevention and Control of Visceral Leishmaniasis in Brazil

**DOI:** 10.1371/journal.pone.0160058

**Published:** 2016-07-29

**Authors:** Anaiá P. Sevá, Fredy G. Ovallos, Marcus Amaku, Eugenia Carrillo, Javier Moreno, Eunice A. B. Galati, Estela G. Lopes, Rodrigo M. Soares, Fernando Ferreira

**Affiliations:** 1 Department of Preventive Veterinary Medicine and Animal Health, School of Veterinary Medicine and Animal Science, University of São Paulo, São Paulo, Brazil; 2 Department of Epidemiology, School of Public Health, University of São Paulo, São Paulo, Brazil; 3 Department of Pathology, School of Medicine, University of São Paulo, São Paulo, Brazil; 4 WHO Collaborating Centre for Leishmaniasis, Centro Nacional de Microbiología, Instituto de Salud Carlos III, Majadahonda, Spain; Instituto Oswaldo Cruz, Fiocruz, BRAZIL

## Abstract

Visceral leishmaniasis (VL) is a zoonosis found worldwide. Its incidence has increased in Brazil in recent years, representing a serious public and animal health problem. The strategies applied in Brazil are questionable and are not sufficient to control the disease. Thus, we have compared the efficacy of some of the currently available strategies focused on dogs to prevent and control zoonotic VL in endemic areas by optimizing a mathematical model. The simulations showed that the elimination of seropositive dogs, the use of insecticide-impregnated dog collars, and the vaccination of dogs significantly contribute to reducing the prevalence of infection in both canines and humans. The use of insecticide-impregnated collars presented the highest level of efficacy mainly because it directly affected the force of infection and vector-dog contact. In addition, when used at a coverage rate of 90%, insecticide-impregnated collar was able to decrease the prevalence of seropositive dogs and humans to zero; moreover, because of the easy application and acceptance by the targeted population, these collars may be considered the most feasible for inclusion in public policies among the three simulated measures. Vaccination and euthanasia were efficacious, but the latter method is strongly criticized on ethical grounds, and both methods present difficulties for inclusion in public policies. When we compared the use of euthanasia and vaccination at coverages of 70 and 90%, respectively, the proportion of infected populations were similar. However, on evaluating the implications of both of these methods, particularly the negative aspects of culling dogs and the proportion of animals protected by vaccination, the latter measure appears to be the better option if the total cost is not significantly higher. The comparison of complications and advantages of different control strategies allows us to analyze the optimal measure and offer strategies to veterinary and public health authorities for making decisions to prevent and control zoonotic VL. Hence, improvements in both public and animal health can be achieved in regions with scenarios similar to that considered in the present study; such scenarios are characteristically found in some areas of Brazil and other countries.

## Introduction

Visceral leishmaniasis (VL) is a widely distributed zoonosis that is found worldwide [1]. In Brazil, 22,491 confirmed human cases and 1,599 deaths occurred from VL from 2007 to 2013 [2], with gradual spreading across all states and a general association with poor living conditions [3]. The primary etiological agent of VL in the Americas is *Leishmania (Leishmania) infantum*, which is mainly transmitted through the bites of infected phlebotomine sandflies [4], particularly the specie *Lutzomyia longipalpis* [5–7]. Infected dogs constitute the main domestic reservoir of the parasite and play a key role in its transmission to humans [8,9].

The current strategies for the prevention and control of VL applied by the Brazilian Health Ministry include 1) early diagnosis and adequate treatment of human cases; 2) use of residual insecticides and sanitary measures targeting the home environment to reduce vector density; and 3) identification and elimination of domestic reservoirs [10]. The elimination of seropositive dogs is increasingly discussed and assessed [11–17], particularly concerning its effect on reducing the prevalence of human and canine disease and its acceptance by the animals’ owners and animal protection institutions [18].

According to Vieira & Coelho [19], approximately 20,000 seropositive dogs are eliminated every year in Brazil. More than 96 million dollars were invested in the program for the control of leishmaniasis from 1988 to 1996, during which more than 150,000 seropositive dogs were euthanized and insecticides were applied in more than one million households [20]. Despite the available resources and the effort invested, the ongoing Brazilian Leishmaniasis Control Program failed to reduce the occurrence of the disease to an acceptable level [3]. Indeed, the prevalence of VL has increased, and the disease has become a serious public health problem in several Brazilian states, indicating that more focused efforts are required [3].

Thus, there is a need for a closer approximation between researchers and public health workers to revise the current control strategies and to define procedures capable of accurately assessing their effects [21]. Alternatively, there are other measures available for prevention and control of VL, which have not yet been implemented on a large scale; these include insecticide-impregnated dog collars, which have been proven efficacious in protecting animals from sandflies [22–25], and Leish-Tec^®^, which is the currently available vaccine for dogs in Brazil. This vaccine has an efficacy of 71%, as determined by the dogs that remain uninfected.[26].

Mathematical models are an important tool used by researchers for the study of the control measures; they can be used to simulate the population dynamics of infectious diseases [27]. An ever-expanding number of diseases and public health questions are being addressed, and the models are able to supply elements useful for the formulation of public policies for the control of diseases [26,27]. Previously developed VL models in Brazil have focused on evaluating prevention and control measures [13,26,27,14,28,29,21]. However, they do not compare all of the currently available measures applied on dogs, and in different coverages, and they do not consider some particular factors, such as an rently available measures atlable measures at an endemica en and dog cases he recovered staget the third analisis the seropomortalitymorrrrmommbothmortality, the repellent effect of the insecticide-impregnated collars or the efficacy of the available vaccines. In addition, they do not include certain characteristics that are necessary to reflect the biological reality of the dynamic disease, such as the clinical-immunologic conditions of the hosts.

This study adapted a model of VL proposed by Burattini et al. [29]; however, we also included distinct values for the the parameters of the forces of infection to the hosts and vector, and we introduced measures that were not previously addressed. Thus, we compared the efficacy of some of the available measures that focus on dogs, to be used in public policy such as deltamethrin-impregnated collars, vaccination and culling, using the VL mathematical model. This study was developed to contribute information to veterinary and public health authorities regarding the current practices of zoonotic VL prevention and control in endemic scenarios, such as in Brazil. Therefore, we have evaluated the optimal measures necessary to considerably decrease the number of human and dog VL cases.

## Materials and Methods

### Model

The mathematical model formulated by Burattini et al. [29] was adapted to allow for the simulation of the control measures. For this purpose, the targeted populations were classified based on their clinical-immune status.

It was assumed that the three targeted populations (humans, dogs and vectors) were constant and that the odds of infection were the same among the various hosts’ age ranges, without seasonal variation.

**Fig 1:**
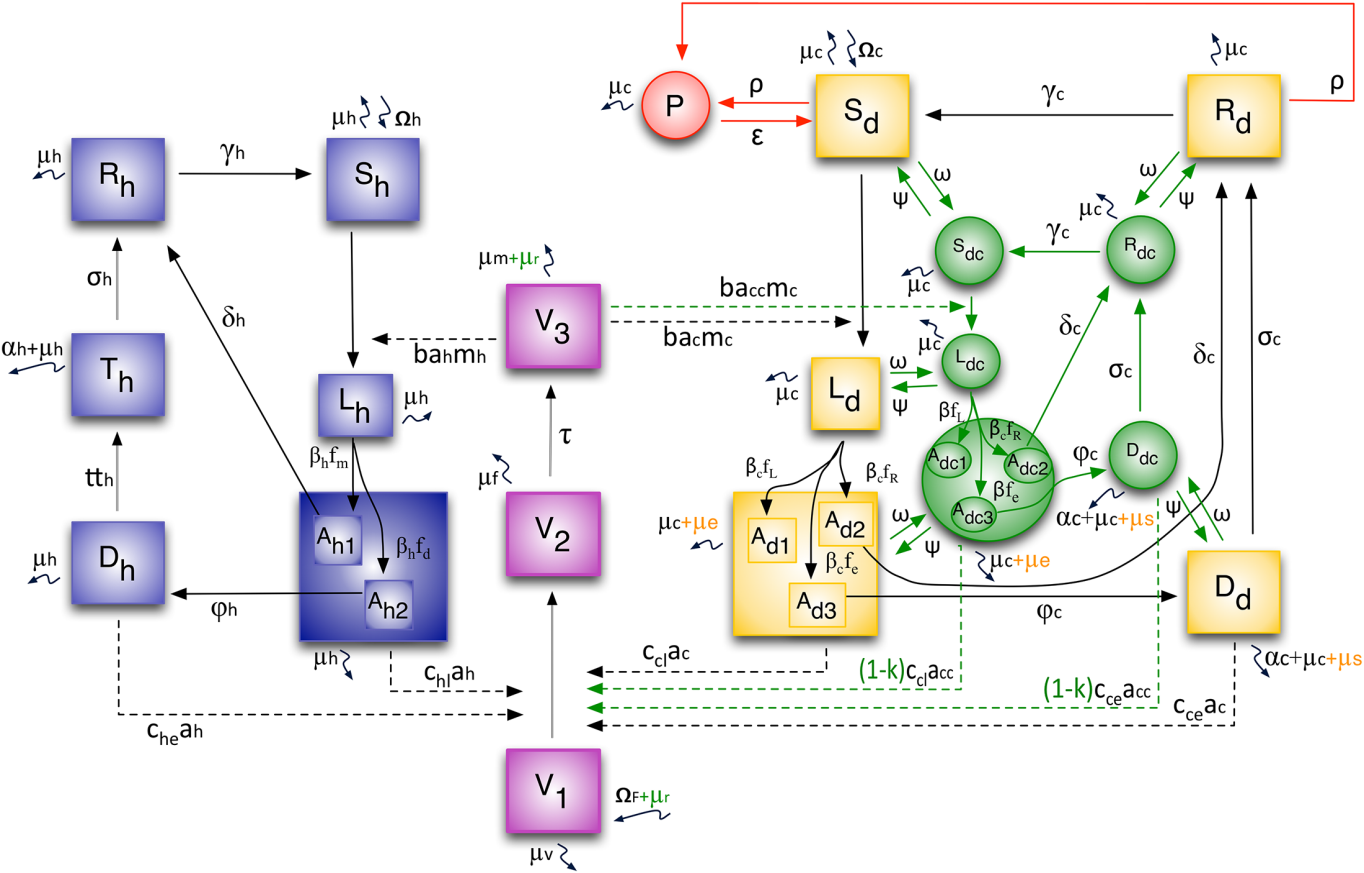
Model of compartments and the flow among them. Legend: blue: human populations; pink: vector populations; yellow: dog populations not subjected to interventions; red: vaccinated dog populations; green: collar-wearing dog populations; orange: mortality by euthanasia.

The disease dynamics in the various populations considering the preventive and control measures are depicted in Fig 1.

The model is described next.

#### Humans

Humans are born susceptible (S_h_) at a rate of **Ω**_**h**_ and are infected at a rate of **ba**_**h**_**m**_**h**_**V**_**3**_, in which **b** is the proportion of infective bites, **a**_**h**_ is the average number of daily vector bites on humans, and **m**_**h**_ is the vector density per human host (eqs 1 and 2). Following infection, humans become infected but are not infective; thus, they enter compartment L_h_. Fraction **f**_**m**_ of the infected, non-infective individuals remains asymptomatic at the compartment A_h1_ until they recover from the disease, and move to that a rate of **β**_**h**_**f**_**m**_, whereas fraction **f**_**d**_ develops symptoms and moves to compartment A_h2_ at a rate of **β**_**h**_**f**_**d**_ (eqs 2–4). The individuals in compartments A_h1_ and A_h2_ are considered to be asymptomatic, and they are discriminated according to their next stage. The value of **β**_**h**_ is given by the inverse of the time an individual remains non-infective and lacks humoral immunity. The infective individuals in compartment A_h1_ become resistant as a function of the development of cell-mediated immunity, and they move to compartment R_h_ at a rate of **δ**_**h**_, whereas the infective individuals in A_h2_ develop disease at a rate of **φ**_**h**_ and move to the compartment of the diseased individuals, D_h_. It was assumed that the diseased individuals are hospitalized and thus move to compartment T_h_ at a rate of **tt**_**h**_, losing their infective capacity. At the hospital, humans may either die from VL at a rate of **α**_**h**_ or recover, whereby they move to compartment R_h_ at a rate of **σ**_**h**_. With the loss of cellular immunity, the recovered individuals become susceptible again, thus moving to compartment S_h_ at a rate of **γ**_**h**_. All humans are subjected to the natural mortality rate **μ**_**h**_ (eqs 1–7).

$$\frac{dS_{h}}{dt}=\;–S_{h}ba_{h}m_{h}V_{3}+\mu_{h}\left(L_{h}+A_{h1}+A_{h2}+D_{h}+T_{h}+R_{h}\right)+T_{h}\alpha_{h}+R_{h}\gamma_{h}$$ (1)

$$\frac{dL_{h}}{dt}=S_{h}ba_{h}m_{h}V_{3}-L_{h}\left(\mu_{h}+(\beta_{h}\left(f_{m}+f_{d}\right))\right)$$ (2)

$$\frac{dA_{h1}}{dt}=L_{h}\beta_{h}f_{m}-A_{h1}(\mu_{h}+\delta_{h})$$ (3)

$$\frac{dA_{h2}}{dt}=L_{h}\beta_{h}f_{d}-A_{h2}(\mu_{h}+\varphi_{h})$$ (4)

$$\frac{dD_{h}}{dt}=A_{h2}\varphi_{h}-D_{h}(\mu_{h}+t_{th})$$ (5)

$$\frac{dT_{h}}{dt}=D_{h}t_{th}-T_{h}(\mu_{h}+\sigma_{h}+\alpha_{h})$$ (6)

$$\frac{dR_{h}}{dt}=A_{h1}\delta_{h}+T_{h}\sigma_{h}-R_{h}(\mu_{h}+\gamma_{h})$$ (7)

#### Dogs

Dogs are born susceptible, S_d_, at a rate of **Ω**_**d**_ and enter compartment L_d_ when they are infected, which occurs at a rate of **ba**_**c**_**m**_**c**_**V**_**3**_, in which **b** is the proportion of infecting bites, **a**_**c**_ is the average number of daily vector bites on dogs, and **m**_**c**_ is the vector density per canine host (eqs 8 and 9). Following infection, dogs are infected but not infective in this compartment; a fraction (**f**_***l***_) of these individuals remain asymptomatic for their entire lives and move to compartment A_d1_, whereas a fraction (**f**_**r**_) remain asymptomatic at the compartment A_d2_ until they recover from the disease. Those that develop symptoms (the fraction **f**_**e**_) move to compartment A_d3_. The individuals in A_d1_, A_d2_ and A_d3_ are considered asymptomatic, and they are discriminated according to their next stage. The development of infectiveness and humoral immunity, with the move of the individuals in compartment L_d_ to compartments A_d1_, A_d2_ and A_d3_, occurs at rates of **β**_**c**_**f**_**l**_, **β**_**c**_**f**_**r**_ and **β**_**c**_**f**_**e**_, respectively (eqs 9–12); the value of **β**_**c**_ is given by the inverse of the time an individual remains non-infective and lacking humoral immunity. Individuals A_d2_ become resistant as a function of the development of cell-mediated immunity, and they move to compartment R_d_ at a rate of **δ**_**c**_, whereas individuals A_d3_ become diseased at a rate of **φ**_**c**,_ thus moving to compartment D_d_. These latter individuals may die from the disease at a rate of **α**_**c**_ or recover (R_d_) at a rate of **σ**_**d**_. The recovered individuals lose their cellular immunity and become susceptible (S_d_) at a rate of **γ**_**d**_ (eq 13). The treatment of diseased animals was not considered. All dogs were subjected to the natural mortality rate **μ**_**c**_ (eqs 8–14).

The prevention and control methods are described next.

**Insecticide-impregnated Collar:** The use of collars as a preventive and control measure was simulated by assuming a rate of application **ω** to all of the dog population compartments, which is applied independent from the clinical-immune status of the animals. The collars have two effects: 1) they inhibit vector bites, and 2) they cause the death of the insects that bite the animals wearing them [23]. The effect of bite inhibition manifests as a reduction of the value of **a**_**c**_, which becomes **a**_**cc**_ (eqs 15, 16, 23 and 24). The mortality resulting from the collar insecticide effect was considered instantaneous; **k** represents the proportion of insects that die after biting a collar-wearing dog. As a result, the rate of infection of insects that bite collar-wearing dogs in compartments A_dc1_, A_dc2_ and A_dc3_ is represented by **c**_**cl**_**a**_**cc**_(**1-k**), whereas the rate corresponding to the insects that bite collar-wearing dogs in compartment D_dc_ is represented by **c**_**ce**_**a**_**cc**_(**1-k**) (see eqs 23 and 24).

After feeding on collar-wearing dogs and eventually infecting them, vectors (V_3_) die at a rate of **μ**_**r**_ as a result of the collar-induced mortality (eq 25). When the efficacy of the collar is lost, at a rate “**ψ”**, the animal that is using it returns to the previous compartment without collars (eqs 15–21). At this model is being considered the immediate replacement of the collars in case of loss and damage.

**Vaccination:** It was assumed that vaccinated animals do not develop infection, and hence do not infect the vector. Vaccination is applied to seronegative, such as susceptible (S_d_), recovered (R_d_) and newly infected non-infective (L_d_) dogs at a rate of **ρ**. This rate corresponds to the percentage of dogs per year that are intended to receive protection; it was calculated by taking the efficacy of the vaccine into consideration (Table 1). The loss of vaccine-induced immunity occurs at a rate of **ε** (eq 22).

**Table 1:** Symbols and biological meanings of the parameters included in the model with the corresponding values and references.

SYMBOLS AND BIOLOGICAL MEANING		VALUES	REFERENCES
**HUMANS**			
**μ**_**h**_	Natural mortality rate	3.69x10^-5^ day^-1^	[62]
**α**_**h**_	VL lethality	1.38x10^-2^ year^-1^	[2]
**a**_**h**_	Average daily human bites by vector	1.4x10^-1^day^-1^	Assumed (based on Ovallos, [63])
**m**_**h**_	Vector density per human	1.07x10^-1^	Assumed (based on Ovallos, 2013—oral communication)
**β**_**h**_	Latency period (L→A)	3.3x10^-2^day^-1^	Estimated (based on Maia et al. [64])
**δ**_**h**_	Recovery rate of asymptomatic individuals (A→R)	9.1x10^-4^ day^-1^	Estimated (based on Badaro et al. [5]; Carvalho et al. [65]; Silva et al.[66])
**f**_**m**_	Proportion of asymptomatic individuals who recover	83x10^-2^	[67]
**φ**_**h**_	Rate of symptom development (A→D)	4.8x10^-3^ day^-1^	Estimated (based on Brazilian Ministery, [10])
**f**_**d**_	Proportion of individuals who become symptomatic	17x10^-2^	[67]
**tt**_**h**_	Treatment rate (T→R)	2.0x10^-2^ day^-1^	[68]
**σ**_**h**_	Recovery rate of treated individuals (D→R)	1.4x10^-3^ day^-1^	Estimated (based on Carvalho et al. [65]; Silva et al.[66]; Alvar [69])
**γ**_**h**_	Loss of cell-mediated immunity (R→S)	5.47x10^-4^ day^-1^	Estimated (based on Badaro et al. [5]; Carvalho et al. [65]; Silva et al.[66]; Alvar [69])
**Ω**_**h**_	Birth rate	**α**_**h**_ + **μ**_**h**_	
**DOGS**			
**μ**_**c**_	Natural mortality rate	9.23x10^-4^ day^-1^	[70]
**α**_**c**_	VL lethality	2.12 year^-1^	[71]
**a**_**c**_	Average daily dog bites by vector	1.4x10^-1^ day^-1^	Assumed (based on Galvis [63])
**m**_**c**_	Vector density per dog	1.94	Galvis, 2013 (oral communication)
**β**_**c**_	Latency period (L→A)	3.3x10^-2^ day^-1^	[64]
**f**_**L**_	Proportion of individuals that remain asymptomatic	22x10^-2^	[72]
**δ**_**c**_	Recovery rate of asymptomatic individuals (A→R)	5.5x10^-3^ day^-1^	Estimated (based on Fisa et al. [73]; Silva et al. [74])
**f**_**R**_	Proportion of asymptomatic individuals that recover	45x10^-2^	[72]
**φ**_**c**_	Rate of development of symptoms (A→D)	1.1x10^-2^ day^-1^	Estimated
**f**_**e**_	Proportion of individuals that become symptomatic	32x10^-2^	[72]
**σ**_**c**_	Recovery rate of diseased individuals (D→R)	2.73x10^-3^ day^-1^	Estimated (based on Garcia et al. [72]; Pozio et al. [71])
**γ**_**c**_	Loss of cell-mediated immunity (R→S)	2.73x10^-3^ day^-1^	Assumed
**Ω**_**c**_	Birth rate	**α**_**c**_ + **μ**_**c**_	
**VECTOR**			
**μ**_**v**_	Life expectancy of non-infected vectors	9.09x10^-2^ day^-1^	[75]
**μ**_**f**_	Life expectancy of infected, non-infective vectors	1.67x10^-1^ day^-1^	[75]
**μ**_**m**_	Life expectancy of infected and infective vectors	2.5x10^-1^ day^-1^	Estimated (based on Kamhawi [76])
**τ**	Extrinsic incubation period	2.0x10^-1^ day^-1^	[76]
**b**	Fraction of infective bites	1.5x10^-1^	Assumed (based on Burattini et al. [29])
**c**_**hl**_	Proportion of insects that acquire infection after biting latent humans	zero	[77]
**c**_**he**_	Proportion of insects that acquire infection after biting diseased humans	1.2 x10^-2^	[77]
**c**_**cl**_	Proportion of insects that acquire infection after biting latent dogs	38.5x10^-2^	[78]
**c**_**ce**_	Proportion of insects that acquire infection after biting diseased dogs	24.7x10^-2^	[78]
**Ω**_**f**_	Birth rate	**μ**_**v**_ + **μ**_**f**_ + **μ**_**m**_ + **μ**_**R**_	
**SYMBOLS OF INTERVENTIONS**			
**μ**_**e**_	Rate of latent dog culling X test sensitivity	Coverage X **S**_**s**_	
**μ**_**s**_	Rate of diseased dog culling X test sensitivity	Coverage X **S**_**a**_	
**ρ**	Rate of dogs protected by vaccination X vaccine efficacy	Coverage X **VE**	
**VE**	Vaccine efficacy	75%	Approximated value of vaccine efficacies of Leish-Tec^®^ [26]
**ε**	Loss of vaccine-induced immunity	2.7x10^-3^ day^-1^	[79]
**ω**	Rate of collar use	Coverage	
**ψ**	Loss of collar effect	2.8x10^-3^ day^-1^	[23]
**a**_**cc**_	Average daily collar-wearing dog bites by vector **a**_**d**_ X (1 –collar repellent effect)	**a**_**d**_ X 10x10^-2^	[23]
**CE**	Average rate of collar repellent effect	90x10^-2^	[23]
**μ**_**R**_	Mortality of insects that bite collar-wearing dogs	**a**_**cc**_ X **k**	
**k**	Average collar-induced vector mortality rate	55x10^-2^	[23]
**S**_**a**_	Test sensitivity in asymptomatic dogs	98x10^-2^	[50]
**S**_**s**_	Test sensitivity in symptomatic dogs	47x10^-2^	[50]

Vaccination does not inhibit the development of disease when dogs are vaccinated during the “immunological window” (L_d_), therefore, when dogs are vaccinated while in compartment L_d_, they do not move to compartment P but remain in the same compartment, where they undergo the natural course of the infection. However, these individuals must be considered in the calculation of the vaccine doses because they are, in fact, vaccinated.

**Euthanasia:** The elimination of asymptomatic (A_d1_, A_d2_, A_d3_, A_dc1_, A_dc2_ and A_dc3_) and symptomatic (D_d_ and D_dc_) seropositive dogs occurs at rates **μ**_**e**_ and **μ**_**s**_, respectively (eqs 10–12, 17–19, 13 and 20, respectively). These rates differ because they take into consideration the fact that the test sensitivity varies according to the animals’ clinical status. In this model we assume that the populations are constants, thus all dogs that die are added to the compartment of susceptibles, include those euthanized (eq 8).

The euthanasia of 70 and 50% of the seropositive dogs indicates that the average times that these animals remain in the environment are theoretically 1.37 and 1.67 years, respectively, for asymptomatic animals, and 0.67 and 1.00 years, respectively, for symptomatic animals. The symptomatic dogs remain in the environment for a shorter amount of time compared to the asymptomatic dogs because of the improved sensitivity of the diagnostic test. These data were calculated as the inverse of the life expectancy.

$$\begin{array}{ll} \frac{dS_{d}}{dt}= & \left[(\mu_{e}+\mu_{c})\left(A_{d1}+A_{d2}+A_{d3}+A_{dc1}+A_{dc2}+A_{dc3}\right)\right]\\ & +\mu_{c}\left(P+S_{dc}+R_{d}+R_{dc}+L_{d}+L_{dc}\right)+[(\mu_{c}+\mu_{s}+\alpha_{c})\left(D_{d}+D_{dc}\right)]\\ & +R_{d}\gamma_{c}+P\varepsilon +\;S_{dc}\psi \;–\;S_{d}\left(\rho +\omega +ba_{c}m_{c}V_{3}\right) \end{array}$$ (8)

$$\frac{dL_{d}}{dt}=S_{d}ba_{c}m_{c}V_{3}+L_{dc}\psi -L_{d}\left(\mu_{c}+\omega +(\beta_{c}(f_{r}+f_{e}+f_{l}\right)))$$ (9)

$$\frac{dA_{d1}}{dt}=L_{d}\beta_{c}f_{l}+A_{dc1}\psi -A_{d1}\left(\mu_{c}+\mu_{e}+\omega \right)$$ (10)

$$\frac{dA_{d2}}{dt}=L_{d}\beta_{c}f_{r}+A_{dc2}\psi -A_{d2}\left(\mu_{c}+\mu_{e}+\omega +\delta_{c}\right)$$ (11)

$$\frac{dA_{d3}}{dt}=L_{d}\beta_{c}f_{e}+A_{dc3}\psi -A_{d3}\left(\mu_{c}+\mu_{e}+\omega +\varphi_{c}\right)$$ (12)

$$\frac{dD_{d}}{dt}=A_{d3}\varphi_{c}+D_{dc}\psi -D_{d}\left(\mu_{c}+\mu_{s}+\omega +\sigma_{c}+\alpha_{c}\right)$$ (13)

$$\frac{dR_{d}}{dt}=A_{d2}\delta_{c}+R_{dc}\psi +D_{d}\sigma_{c}-R_{d}\left(\mu_{c}+\omega +\rho +\gamma_{c}\right)$$ (14)

$$\frac{dS_{dc}}{dt}=R_{dc}\gamma_{c}+S_{d}\omega –\;S_{dc}\left(\mu_{c}+\psi +ba_{cc}m_{c}V_{3}\right)$$ (15)

$$\frac{dL_{dc}}{dt}=S_{dc}ba_{cc}m_{c}V_{3}+L_{d}\omega -L_{dc}\left(\mu_{c}+\psi +(\beta_{c}(f_{r}+f_{e}+f_{l}\right)))$$ (16)

$$\frac{dA_{dc1}}{dt}=L_{dc}\beta_{c}f_{l}+A_{d1}\omega -A_{dc1}\left(\mu_{c}+\mu_{e}+\psi \right)$$ (17)

$$\frac{dA_{dc2}}{dt}=L_{dc}\beta_{c}f_{r}+A_{d2}\omega -A_{dc2}\left(\mu_{c}+\mu_{e}+\psi +\delta_{c}\right)$$ (18)

$$\frac{dA_{dc3}}{dt}=L_{dc}\beta_{c}f_{e}+A_{d3}\omega -A_{dc3}\left(\mu_{c}+\mu_{e}+\psi +\varphi_{c}\right)$$ (19)

$$\frac{dD_{dc}}{dt}=A_{dc3}\varphi_{c}+D_{d}\omega -D_{dc}\left(\mu_{c}+\mu_{s}+\psi +\sigma_{c}+\alpha_{c}\right)$$ (20)

$$\frac{dR_{dc}}{dt}=A_{dc2}\delta_{c}+R_{d}\omega +D_{dc}\sigma_{c}-R_{dc}\left(\mu_{c}+\psi +\gamma_{c}\right)$$ (21)

$$\frac{dP}{dt}=\rho \left(S_{d}+R_{d}\right)-P\left(\mu_{c}+\varepsilon \right)$$ (22)

#### Vector

The vector insects are born susceptible (V_1_) at a rate of **Ω**_**f**_ and become infected upon biting infective individuals (A_h1_, A_h2_, D_h_, A_d1_, A_d2_, A_d3_ and D_d_) at a rate that depends on the fraction of insects that acquires infection after a bite (**c**_**hl**_, **c**_**he**_, **c**_**cl**_ and **c**_**ce**_, respectively) and the average number of bites on human (**a**_**h**_) or dog (**a**_**c**_) hosts per day (eqs 24 and 25). These parameters differ because they vary in each population. The infected insects (V_2_) become infective (V_3_) at a rate of **τ**, which is defined by the extrinsic incubation period. The vector mortality rate is given by **μ**_**v**_, **μ**_**f**_ and **μ**_**m**_ for each of its phases (V_1_, V_2_ and V_3_, respectively) (eqs 23–25).

$$\frac{dV_{1}}{dt}=V_{3}\left(\mu_{r}+\mu_{m}\right)+V_{2}\mu_{f}-V_{1}\left[c_{hl}a_{h}\left(A_{h1}+A_{h2}\right)+c_{he}a_{h}D_{h}+c_{cl}a_{c}\left(A_{d1}+A_{d2}+A_{d3}\right)+c_{cl}a_{cc}\left(1-\kappa \right)\left(A_{dc1}+A_{dc2}+A_{dc3}\right)+c_{ce}a_{cc}\left(1-\kappa \right)D_{dc}+c_{ce}a_{c}D_{d}\right]$$ (23)

$$\frac{dV_{2}}{dt}=V_{1}\left[c_{hl}a_{h}\left(A_{h1}+A_{h2}\right)+D_{h}c_{he}a_{h}+c_{cl}a_{c}\left(A_{d1}+A_{d2}+A_{d3}\right)+c_{cl}a_{cc}\left(1-\kappa \right)\left(A_{dc1}+A_{dc2}+A_{dc3}\right)+c_{ce}a_{cc}\left(1-\kappa \right)D_{dc}+c_{ce}a_{c}D_{d}\right]-V_{2}\left(\tau +\mu_{f}\right)$$ (24)

$$\frac{dV_{3}}{dt}=V_{2}\tau -V_{3}\left(\mu_{m}+\mu_{r}\right)$$ (25)

### Model parameters

Some of the parameters that we used were obtained from studies performed in the city of Panorama, São Paulo, Brazil, and others were estimated or adjusted to represent the actual situation in that city (Table 1). The canine seroprevalence in that municipality between August 2012 and February 2013 was 25% (LOPES et al., non-published). This region represents a scenario of some visceral leishmaniasis endemic areas in Brazil, and with similar conditions according to the public health system, with low-income, and political structure.

### Simulated scenarios

The three preventive and control measures were simulated with 70 and 90% coverage. The simulations were performed using Matlab version R2013a.

### Sensitivity of the model

**Fig 2:**
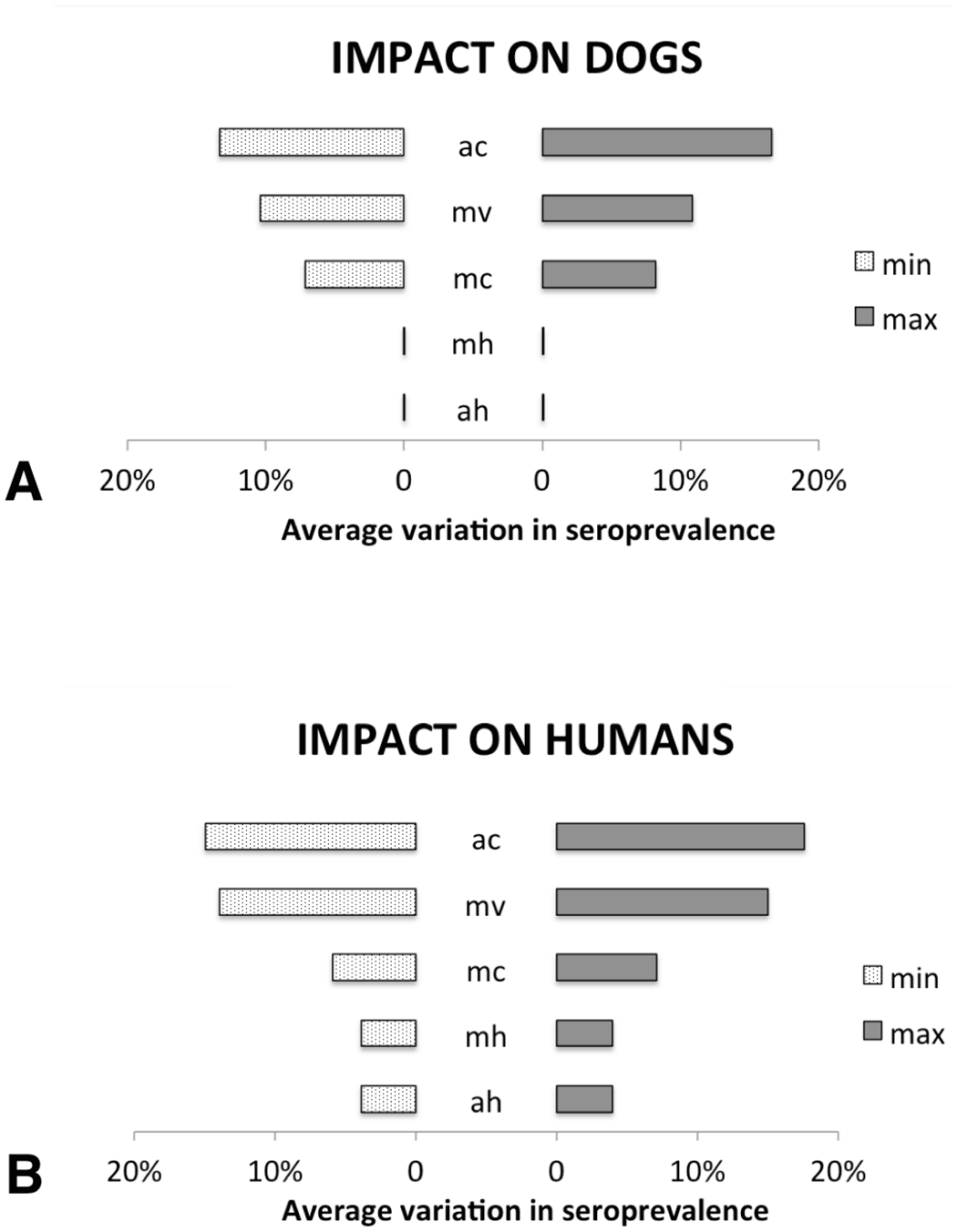
Effect of the parameter variations in the host seroprevalences. Average variations in dog (A) and human (B) seroprevalences according to the maximum (max) and minimum (min) values of the parameter variations. Legend: ah) average daily human bites by the vector; ac) average daily dog bites by the vector; mh) density of the vector per human; mc) density of the vector per dog; mv) life expectancy of the infected vectors (muf and mum).

To evaluate the sensitivity of the model parameters, the density of the vector per host, the average daily hosts by the vector, and the life expectancy of the infected vectors were varied by 0.5%, 1%, 4%, 7% and 10%. Thus, the average seroprevalence variations of human and dog populations for each parameter were calculated and are presented in Fig 2.

## Results

### Simulation of control measures

The results of the simulations were plotted in graphs representing the dog, human and vector populations as a function of time. These populations were estimated from the time that they reached a stable equilibrium to allow the comparison of the measures applied, as Table 2 shows.

**Table 2:** Proportions of the human, dog, and vector populations.

Intervention	Cover	HUMANS					DOGS					VECTORS	
		Asympt (L+A)	Sick (D)	Recov	Protec	Cover	Susc	Asympt (L+A)	Sick (D)	Recov	Protec	V1	V2+V3
WITHOUT		25.0%	2.13%	8.8%	0.0%		86.3%	25.0%	2.13%	8.8%	0.0%	93.99%	6.01%
VAC	90%	3.1%	0.24%	0.5%	44.2%	90%	96.7%	3.1%	0.24%	0.5%	44.2%	99.20%	0.80%
	70%	6.9%	0.57%	1.5%	33.1%	70%	94.6%	6.9%	0.57%	1.5%	33.1%	98.25%	1.75%
COLL	90%	0.2%	0.01%	0.0%	0.0%	90%	99.3%	0.2%	0.01%	0.0%	0.0%	99.97%	0.03%
	70%	1.4%	0.10%	0.4%	0.0%	70%	98.4%	1.4%	0.10%	0.4%	0.0%	99.76%	0.24%
EUT	90%	0.3%	0.02%	0.2%	0.0%	90%	99.0%	0.3%	0.02%	0.2%	0.0%	99.92%	0.08%
	70%	2.2%	0.20%	1.0%	0.0%	70%	97.6%	2.2%	0.20%	1.0%	0.0%	99.47%	0.53%

Impregnated-insecticide collar (COLL), vaccination (VAC) and euthanasia (EUT) according to the coverage (cover.) rates. For humans and dogs: Susc: susceptible; Asympt: asymptomatic (L: latent and A: asymptomatic); sick (D: with symptoms); Recov: recovered; T: humans in treatment; R: hunans recovered; Protec: dogs protected by the vaccine. For vectors: V1: non-infected; V2: infected but not infective; V3: infective.

The measures associated with the lowest proportions of humans and dogs in the infective stages and infected insects were considered the most efficacious measures because they indicated that the prevalence of VL was reduced.

The seroprevalences in Table 2 show that all of the simulated measures had significant favorable effects on the dog and human populations, i.e., the proportions of infected dogs and humans in both the symptomatic and asymptomatic stages decreased substantially until attaining stable equilibrium. The effect of the collar was more intense than that of euthanasia and vaccination for these infected populations. The proportion of asymptomatic dogs and humans prior to the intervention were 25% and 4.81%, respectively; these values decreased by approximately fifteen and ten times, reaching less than 1.5% and 0.4%, respectively, when the collar was used at a coverage rate of 90%. When 90% of the seropositive dogs were culled, these values decreased by approximately ten and eight times, reaching less than 2.5% and 0.6%, respectively, and vaccination of 90% of the seronegative dogs caused these values to decrease by approximately four and three times, reaching less than 7% and 1.7%, respectively.

A 90% coverage rate of the insecticide-impregnated collar resulted in eliminating the sick population of humans and reducing the number of sick dogs to nearly zero percent. Euthanizing 90% of the seropositive dogs decreased the number of sick animals to nearly zero (0.02%) as well, and vaccination reached a rate of 0.24%.

The proportion of susceptible individuals increased significantly, reaching nearly one hundred percent when using the collar and culling in 70% and 90% of the population, respectively. Conversely, with the use of vaccination, the proportion of susceptible dogs was the lowest with respect to the use of other measures and when no measures were used. However, the protected animals represented a significant proportion of the population when the seronegative dogs were vaccinated at rates of 70 or 90%.

The variation in the coverage rates of all three simulated measures, ranging from 70 to 90%, strongly affected the prevalence rates generated in the dog and human populations. When the coverage rate of the collar increases from 70 to 90%, the proportion of sick dogs decreases ten times (from 0.10 to 0.01%), and the proportion of asymptomatic human decreases by five times (from 0.31 to 0.06%). When the coverage of the vaccine increases from 70 to 90%, the proportion of sick dogs and humans is nearly cut in half, from 0.57 to 0.24% and from 0.014 to 0.007%, respectively, and the proportion of asymptomatic dogs and humans is also reduced from 6.9 to 3.1% and from 1.65 to 0.87%, respectively.

The scenario of infected humans and dogs (sick and asymptomatic) is similar when using a vaccination coverage rate of 90% and a euthanasia rate of 70%; however, euthanasia does not result in a protected population.

### Sensitivity of the model

Variations in the amplitude of the dog and human seroprevalences highlight that the most sensitive parameters are those that represent the vector contact with dogs, in particular the average daily bites by the vector. The life expectancy of the infected vectors is also sensitive in this model. Both the average daily bites by the vector in humans bitten (a_h_) and the density of the vector per human (m_h_) parameters present a similar behavior: they were less sensitive and affected just the infected human population.

## Discussion and Conclusion

All of the control measures applied to the dogs, in the different coverages, were associated with a decrease in the prevalence of infection in the human population, indicating the importance of the infected dog population in the occurrence of VL in humans. Similarly, Burattini et al. [29] (based on a mathematical model) and Nunes et al. [30] observed that the establishment of this disease as endemic among humans is highly dependent on its prevalence among the dog population.

Among the control measures simulated in the present study, the deltamethrin-impregnated collar was associated with the most significant effects. The decrease in asymptomatic dogs and humans by approximately fifteen and ten times, respectively, after the use of the collar in 90% of the dogs represents a large number of individuals in the environment. Thus, it is worth noting that the simulation of collar use encompassed a larger number of animals because it included both the seronegative and seropositive dogs. Such observations were also made by Reithinger et al. [25], who stated that the proportion of dogs wearing collars must be quite high to achieve a significant reduction of VL prevalence. In this regard, the application of collars to dogs that replace others in a given population and the rapid replacement of collars are considered crucial because the rate of collar loss or damage, can vary among 4.9% (1,796/36,638) [15], and 23% (289/1,246) [31] or 0.006 per day [25]. In our model we considered the immediately replacement of these collars, which implies in permanent surveillance to ensure the continuous protection as suggested by Oliveira-Lima et al. [31].

The efficacy of collars as a method for the control of VL is a result of two factors. The first is associated with its insecticide effect, in which the number of newly infected insects (represented by compartment V3) decreases because a significant fraction of them die after its contact with the dogs. This effect was also demonstrated indirectly in other studies that simulated the use of insecticides and found a substantial reduction in the disease prevalence in and humans. In another study with topic insecticides, it was observed that it reduced the probability of introduction and establishment of canine leishmaniasis [32]. The other factor concerns the repellent effect of the collar, which manifests as reductions in the average number of bites per day (**a**_**c**_ and **a**_**h**_), consequently reducing the force of infection and the rate of infected insects. However, the repellent effect may hinder the insects from biting collar-wearing animals and make them bite other reservoirs.

These two factors are associated with the more sensitive parameters in the model, which include those related to vector contact with dogs and the mortality rates of infected vectors; thus, this measure significantly affects the leishmaniasis dynamic.

The large-scale use of deltamethrin-impregnated dog collars over two years in Italy promoted a reduction of 50% in the incidence of canine VL after the first year and 98% after the second year in one study [33]; another study showed a reduction of 83% after one year [34]. In a study conducted in Iran, the risk of seroconversion after infection with *Leishmania* spp. decreased among collar-wearing dogs, and the seroprevalence of disease decreased among children [24]. Similarly, the application of collars to 88% of the dogs in Andradina, São Paulo, Brazil, over two years reduced the seroprevalence of canine disease from 10.8 to 4.8% and decreased its incidence among humans [15]. In the present study, the simulated application of collars to 90% of the canine population induced a higher and strong reduction of the seroprevalence among both dogs and humans, from 27.1 to 0.2% and from 4.85 to 0.06%, respectively, which agrees with the empirical data.

The possible occurrence of allergic dermatitis in some of the animals must be considered relative to the large-scale use of collars. In a study by Camargo-Neves et al. [15], the proportion of affected animals was 2.3% (832/36,638). Some cases of dermatitis require treatment; thus, monitoring the animals and providing them with veterinary care are highly important. In addition, the application of collars to 70 or 90% of the dogs in a population, as was simulated in the present study, requires intense effort. Although this strategy does not require previous serologic tests, these coverages correspond to a high number of dogs, because these rates refer to the total population (i.e., seropositive and seronegative animals), and the collars must be replaced every six months or immediately in case of loss and damage.

The *per capita* incidence of a disease in a population is known as the force of infection, and it can be reduced through vaccination, thus making individuals pass from the status of susceptible to resistant while by-passing the state of infection [35]. According to their efficacy, vaccines may induce the full elimination of an agent by reducing the number of secondary cases [35]. Foroughi-Parvar & Hatam [36] state that vaccines are the best choices to access a convenient and efficacious method for the control of zoonotic VL.

The efficacy of vaccination depends on the coverage rate. Using a mathematical model, Dye [27] confirmed that when a vaccination has 100% efficacy, it is more effective than culling; however, the proportions of animals subjected to vaccination and culling in his study were not made clear. Dye further observed that at the time the study was performed, there were no vaccines with 100% efficacy, which is still the case. The CaniLeish^®^ vaccine, which is available in Europe, displayed similar efficacy (70%) to Leish-Tec^®^, as used in this study. According to Gradoni et al. [37], studies on vaccines for leishmaniasis are still in need of impetus to develop efficacious and universally applicable vaccines. However, in recent decades, major research has been conducted to innovate new vaccines [36].

In our study, we simulated a vaccine with an efficacy of 75%. Using a higher efficacy in the simulations would result in better outcomes and would also decrease the number of animal subjects to infection, resulting in a low force of infection to susceptible dogs, which significantly influences the disease dynamic, as observed in the sensitivity analysis of the parameter.

The simulated vaccination of dogs in the present study substantially reduced the proportion of infected humans, as was also observed in the theoretical study by Dye [27]. Reis et al. [38] affirmed that the induction of a protective anti-*Leishmania* immunity response in dogs is a feasible, important, and cost-effective goal that highly affects the control of human leishmaniasis [27]. Palatnik-de-Sousa et al. [39] analyzed studies on dog vaccination conducted in Brazil and found that the reduction of the prevalence of canine and human disease was directly related to the increase in the number of vaccinated dogs.

From an epidemiological viewpoint, the main goal is to reduce the number of non-infected and non-infective animal and human hosts and therefore eliminate the disease agent from the environment. The vaccine was able to considerably decrease the number of infected hosts and was still able to make a large proportion of the protected animals susceptible, which is a great advantage.

In the present model we simulated data about the available vaccine in Brazil (Leishtec^®^). Although this vaccine presents an importance efficacy a recent study observed local and systemic adverse effects in 13% (6/46) of the vaccinated animals [40]. Therefore, these adverse effects represent a potential limitation of this strategy since it may affect compliance and might require veterinary assistance.

Although Marzochi et al. [41] suggest that vaccination is an efficient control measure and recommend its application concomitantly to the anti-rabies vaccination program, some considerations should be mentioned: 1) the efficacy of the vaccines has not yet been clearly established, 2) their individual cost is high, and 3) the application regimen involves three initial doses and demands previous serologic testing. Furthermore, adding the leishmaniasis vaccine to the anti-rabies vaccination program requires that pet owners take their dogs to a vaccination point, which can be a logistic complication because endemic areas are normally associated with low-income regions [42,43,44,45,46]. Consequently, it is difficult to establish vaccination as a public policy.

The euthanasia of seropositive dogs was efficacious in the present study, to reduce the prevalence of the infection. However, it requires a significant sampling effort and its application is strongly criticized on ethical grounds, with resistance from the population of the dog owners and workers who perform this function. Particularly one of the difficulties of euthanize practice refers to the elimination of asymptomatic infected dogs, because this activity could produce psychological impacts on veterinary medicals by the constant demands to eliminate healthy animals and are in face to moral stress, as pointed by Rollin [47].

The results reported here corroborate those of other studies conducted in Brazil, which also demonstrated the efficacy of the measure, with repercussions on both canine and human populations. In Teresina, Piauí, Werneck et al. [48] assessed the use of an insecticide and the culling of dogs and found that the latter had a significantly higher efficacy compared to the control group. The culling of 48% of the dogs was associated with a reduction in the prevalence of human disease 18 months later. In Jacobina, Bahia, Ashford et al. [12] found that after four years of euthanatizing all seropositive dogs, the canine seroprevalence decreased from 36 to 6% and the incidence of human disease was reduced from ten to two cases among children per year in the intervention area, whereas in the control area, the canine seroprevalence varied from 24 to 28%. In Araçatuba, São Paulo, a negative correlation was also found between the culling of seropositive dogs and the prevalence of human disease; in addition, the results showed that the incidence increased after the intervention was interrupted [17]. In four states in Northeastern Brazil, where the incidence of disease is high, the number of human cases also increased when the culling of dogs was discontinued [13].

According to Courternay et al. [14], euthanasia leads to a significant reduction in the proportion of infective dogs to almost zero, provided by using a diagnostic test with high sensitivity and by eliminate the animals immediately after the diagnosis. Similarly, the use of low-sensitivity serologic tests maintains active reservoirs in endemic areas, which does not reduce the levels of human infection [49]. According to Grimaldi et al. [50], the tests currently used in governmental campaigns, the sensitivity of the screening test is less than 50% among the asymptomatic animals and 98% among the symptomatic ones. In contrast, in a study by Laurenti et al. [51], the test sensitivity was significantly higher in both the asymptomatic and symptomatic animals (92.1 and 89.4%, respectively), and the combination of this test with another attained a sensitivity of 99.1%. In the present study, the sensitivity of the diagnostic test in the asymptomatic animals was simulated to be less than 50%; nevertheless, a significant reduction in the proportion of infective animals was detected after culling.

The application of a mathematical model formulated by Costa et al. [16] assessed the success of a control program involving the euthanasia of symptomatic dogs in areas with low transmission rates (canine seroprevalence = 3%) and in areas with high transmission rates (canine prevalence = 12%), in which both the asymptomatic and symptomatic dogs were euthanized. These data agree with the results of the present study regarding the program success in areas with a high number of dog cases.

In a study by Moreira et al. [52], euthanasia neither increased nor decreased the prevalence of canine disease. That study lasted for two years at a location where the prevalence of canine disease was 31% (Jequié, Bahía) and involved a rather rapid elimination of the seropositive animals up to 14 days after a serodiagnosis was established.

Conversely, although the euthanasia has been applied in Brazil for several years, the number of cases of the disease in the country increased, particularly in urban areas, meaning that this measure was not efficacious [21]. Using a mathematical model, Dye [27] found that euthanasia was the worst option to control disease and was associated with only a small reduction in the human disease prevalence. Moreno & Alvar [8] suggested that the incomplete coverage of seropositive dogs is one reason highlighting the lack of success of this measure. In the present study, the elimination of 70% of the seropositive dogs per year was an efficacious simulated measure, but seropositive dogs and humans still remain in the environment.

Some studies suggest that a significant reduction in the prevalence of canine cases may not occur because of the high dog population turnover rate [53], along with an increasingly younger and thus more disease-susceptible population [30] that can be rapidly infected with the parasite [27]. In towns where the euthanasia of dogs seropositive for VL is performed as a control measure, such as in Araçatuba, São Paulo, from 2002 to 2004, dog turnover occurred in 38.8% (202/521) of the households, and the mean age of dogs was seven months [30]. In another study, 44.5% of the dogs euthanized in 2004 were replaced [54]. In Panorama, São Paulo, 41.5% (496/1,194) of the canine population was two years old or younger, a reflection of the replacement because of compulsory euthanasia [55]. In the model in the present study, the animals that died from euthanasia or VL lethality were born susceptible because that population is constant; nonetheless, the disease prevalence decreased substantially after simulated euthanasia.

Some studies have reported that long delays between diagnosis and euthanasia impair the success of this control measure [40,3,8], partly because the confirmation test may occur in laboratories far from certain municipalities and because of the resistance of the population. This is observed in many large and small endemic municipalities of Brazil, presenting a difficult question to solve.

A euthanasia rate of 70% and a vaccination rate of 90% result in similar outcomes in the infected dogs and humans. However, in view of the implications associated with both measures—the negative features of culling dogs and the proportion of protected animals resulting from the vaccination—the latter method appears to the better option. In addition, it is important to consider the total cost of each measure. The number of animals that must be vaccinated based on the proportion of seronegative dogs is higher than the number that would require euthanasia, which is based on the proportion of seropositive dogs (around 25%). The financial costs of implementing vaccination can be high; however, euthanasia is associated with various disadvantages and difficulties, as previously discussed.

The present model was implemented according to data available in the literature, except for parameters related to the vectorial capacity, such as density of the vector per host and the rate of vectors becoming infected after biting infected hosts. These data are difficult to obtain due to sensitivity of the vector to environmental factors. As cited by EFSA and Cameron et al. [32,56], more information is needed about the sand fly population and their behavior. Thus, it was necessary to fit the parameters, based on the available data, to produce a model that represented the scenarios of VL endemic areas. However, although these parameters were identified as sensitive in the model, this fact does not negate the result’s validation, and it can be applied to regions with similar scenarios. This is the case in some municipalities of the West region of São Paulo State; the canine seroprevalence has reached 30% in Santa Mercedes [57], and in other areas of the country, such as Natal-RN, Cuiabá-MT, Jequié-BA, and Rondônia the seroprevalence was 32% [58], 22%[59], 31% [60], and 28% [61], respectively. In areas with smaller transmission rates, other control measures may produce different results, as observed by Costa et al. [16], who noted that the sensitive parameters related to the vector-dog contact could influence the results substantially.

For a VL control program to be successful, it is necessary to take into account both the efficacy and the feasibility of the measures applied and their acceptance by the targeted population. Based on the strategies simulated in the present study and the socio-economic structure in developing countries, the use of insecticide-impregnated dog collars appears to be the most viable option from an operational point of view. Although this strategy demands a high rate of coverage relative to the total canine population (asymptomatic and symptomatic animals) and replacement of the collars every six months, its advantages include acceptance by the targeted population, simple application at the home of the pet owner, no requirement for previous serologic testing, and efficacy in reducing the seroprevalence among dogs and humans. However, careful attention is required to observe the development of side effects and for replacing the lost or damaged collars.

The implementation of more than one control measure for zoonotic VL used together should present a good efficacy, however the use of only one measure is focused on easily and practicality in terms of instruction for employees, financial resources management, and on the feasibility of operating capacity. Thus, the suggestion of the comparison of scenarios measures applied of isolated form using theoretical studies contribute as a tool in the orientation for making decision by public institutions to prevent and control the disease.

For control measures to be effective, they must be applied on a continuous basis, as shown in our simulations. According to Camargo-Neves et al. [15], currently, the greatest problem in areas where VL is endemic in Brazil is the lack of continuity of preventive and control measures. Furthermore, longitudinal epidemiological and entomological observations during interventions are essential [56]. Hence, lower costs and fewer obstacles in the application of the strategy contribute to its better continuity.

In conclusion, we have developed a mathematical tool that compares different scenarios for implementation of available preventive measures for visceral leishmaniasis, taking into account the disease dynamics in dogs and humans, the distinct capacities of the vector to infect and to be infected by these hosts, the sensitivity of serologic tests, and vaccine and collar efficacies. This model can be applied to other endemic regions by using their epidemiologic data and their respective available prevention and control measures.

In summary, the measures evaluated in this study, when used at high coverage rates, showed significant reduction in number of infected humans (near zero) and also in the number of infected dogs. The use of insecticide-impregnated collars was found to be the optimal measure due to its advantages over the other measures; however, it does require an intense effort because a high coverage rate must be achieved relative to the total canine population. The theoretical exercise performed using simulated scenarios, with measures implemented at different coverage rates, was utilized to compare the capacity for decreasing the VL seroprevalence of humans and dogs. Hence, this data contributes to the aim of improving both public and animal health by elucidating the impact of using these measures on a large scale. Nevertheless, it is important to reinforce that the control of this zoonotic disease requires the cooperation of the population and public health authorities, with an integrated approach that includes education, animal welfare principles, vector control, and environment management.
