# Correction: Canine-Based Strategies for Prevention and Control of Visceral Leishmaniasis in Brazil

**DOI:** 10.1371/journal.pone.0162854

**Published:** 2016-09-08

**Authors:** Anaiá P. Sevá, Fredy G. Ovallos, Marcos Amaku, Eugenia Carrillo, Javier Moreno, Eunice A. B. Galati, Estela G. Lopes, Rodrigo M. Soares, Fernando Ferreira

The third author's name is spelled incorrectly. The correct name is: Marcos Amaku. The correct citation is: Sevá AP, Ovallos FG, Amaku M, Carrillo E, Moreno J, Galati EAB, et al. (2016) Canine-Based Strategies for Prevention and Control of Visceral Leishmaniasis in Brazil. PLoS ONE 11(7): e0160058. doi:10.1371/journal.pone.0160058
